# A novel method for extracting nucleic acids from dried blood spots for ultrasensitive detection of low-density *Plasmodium falciparum *and *Plasmodium vivax *infections

**DOI:** 10.1186/s12936-017-2025-3

**Published:** 2017-09-18

**Authors:** Kayvan Zainabadi, Matthew Adams, Zay Yar Han, Hnin Wai Lwin, Kay Thwe Han, Amed Ouattara, Si Thura, Christopher V. Plowe, Myaing M. Nyunt

**Affiliations:** 1 Division of Malaria Research, Institute for Global Health, University of Maryland School of Medicine, Baltimore, USA; 2 Department of Medical Research, Ministry of Health and Sports, Yangon, Myanmar; 3 Community Partners International, Yangon, Myanmar

**Keywords:** Malaria, Malaria elimination, *Plasmodium falciparum*, *Plasmodium vivax*, Ultrasensitive PCR, Limits of detection, Molecular surveillance, Asymptomatic infection, Diagnostics, Myanmar, Southeast Asia, Dried blood spot, DBS, Low transmission

## Abstract

**Background:**

Greater Mekong Subregion countries are committed to eliminating *Plasmodium falciparum* malaria by 2025. Current elimination interventions target infections at parasite densities that can be detected by standard microscopy or rapid diagnostic tests (RDTs). More sensitive detection methods have been developed to detect lower density “asymptomatic” infections that may represent an important transmission reservoir. These ultrasensitive polymerase chain reaction (usPCR) tests have been used to identify target populations for mass drug administration (MDA). To date, malaria usPCR tests have used either venous or capillary blood sampling, which entails complex sample collection, processing and shipping requirements. An ultrasensitive method performed on standard dried blood spots (DBS) would greatly facilitate the molecular surveillance studies needed for targeting elimination interventions.

**Methods:**

A highly sensitive method for detecting *Plasmodium falciparum* and *P. vivax* 18S ribosomal RNA from DBS was developed by empirically optimizing nucleic acid extraction conditions. The limit of detection (LoD) was determined using spiked DBS samples that were dried and stored under simulated field conditions. Further, to assess its utility for routine molecular surveillance, two cross-sectional surveys were performed in Myanmar during the wet and dry seasons.

**Results:**

The lower LoD of the DBS-based ultrasensitive assay was 20 parasites/mL for DBS collected on Whatman 3MM filter paper and 23 parasites/mL for Whatman 903 Protein Saver cards—equivalent to 1 parasite per 50 µL DBS. This is about 5000-fold more sensitive than standard RDTs and similar to the LoD of ≤16–22 parasites/mL reported for other ultrasensitive methods based on whole blood. In two cross-sectional surveys in Myanmar, nearly identical prevalence estimates were obtained from contemporaneous DBS samples and capillary blood samples collected during the wet and dry season.

**Conclusions:**

The DBS-based ultrasensitive method described in this study shows equal sensitivity as previously described methods based on whole blood, both in its limit of detection and prevalence estimates in two field surveys. The reduced cost and complexity of this method will allow for the scale-up of surveillance studies to target MDA and other malaria elimination interventions, and help lead to a better understanding of the epidemiology of low-density malaria infections.

**Electronic supplementary material:**

The online version of this article (doi:10.1186/s12936-017-2025-3) contains supplementary material, which is available to authorized users.

## Background

The emergence of multidrug resistant *Plasmodium falciparum* malaria has led to a regional malaria elimination campaign in the Greater Mekong Subregion (GMS) [1–8]. Recent studies have found that many malaria infections in GMS are at densities below the level of detection of standard rapid diagnostic tests (RDTs), and do not cause sufficient symptoms to lead to drug treatment [9–11]. This large, clinically silent pool of previously unrecognized malaria is potentially worrisome as it may serve as an important transmission reservoir, complicating ongoing elimination efforts [10–15]. Eliminating *P. falciparum* malaria by 2025 may require clearing both symptomatic and asymptomatic infections.

While well-known to be common in Africa [16–18], the extent of asymptomatic malaria in the GMS was not recognized until diagnostic tests sensitive enough to identify the very low parasite densities associated with such infections were developed [19, 20]. These ‘ultrasensitive’ polymerase chain reaction (usPCR) techniques are thousands of times more sensitive than RDTs and microscopy, and tens-to-hundreds of times more sensitive than even standard PCR [21–24]. Prevalence surveys using usPCR have confirmed that the vast majority of malaria infections in the GMS are subpatent and clinically silent [9, 10, 21].

Despite their high sensitivity, the utility of ultrasensitive techniques has been limited by their cost and complexity, including the need to collect and transport whole blood. Simpler, cheaper and more field-friendly techniques will be required for usPCR to become a useful tool for routine malaria surveillance. Recently, an improved ultrasensitive method was described that requires only 0.3 mL of capillary blood obtained by finger- or ear-prick, with no need for refrigeration for up to 14 days [21]. This report describes further progress toward a scalable molecular surveillance tool through development of a novel extraction method for standard dried blood spots (DBS) that allows ultrasensitive detection of *P. falciparum* and *P. vivax* asymptomatic infections with equal sensitivity as techniques that use whole blood.

## Methods

### Optimization of nucleic acid extraction from dried blood spots

**Fig. 1:**
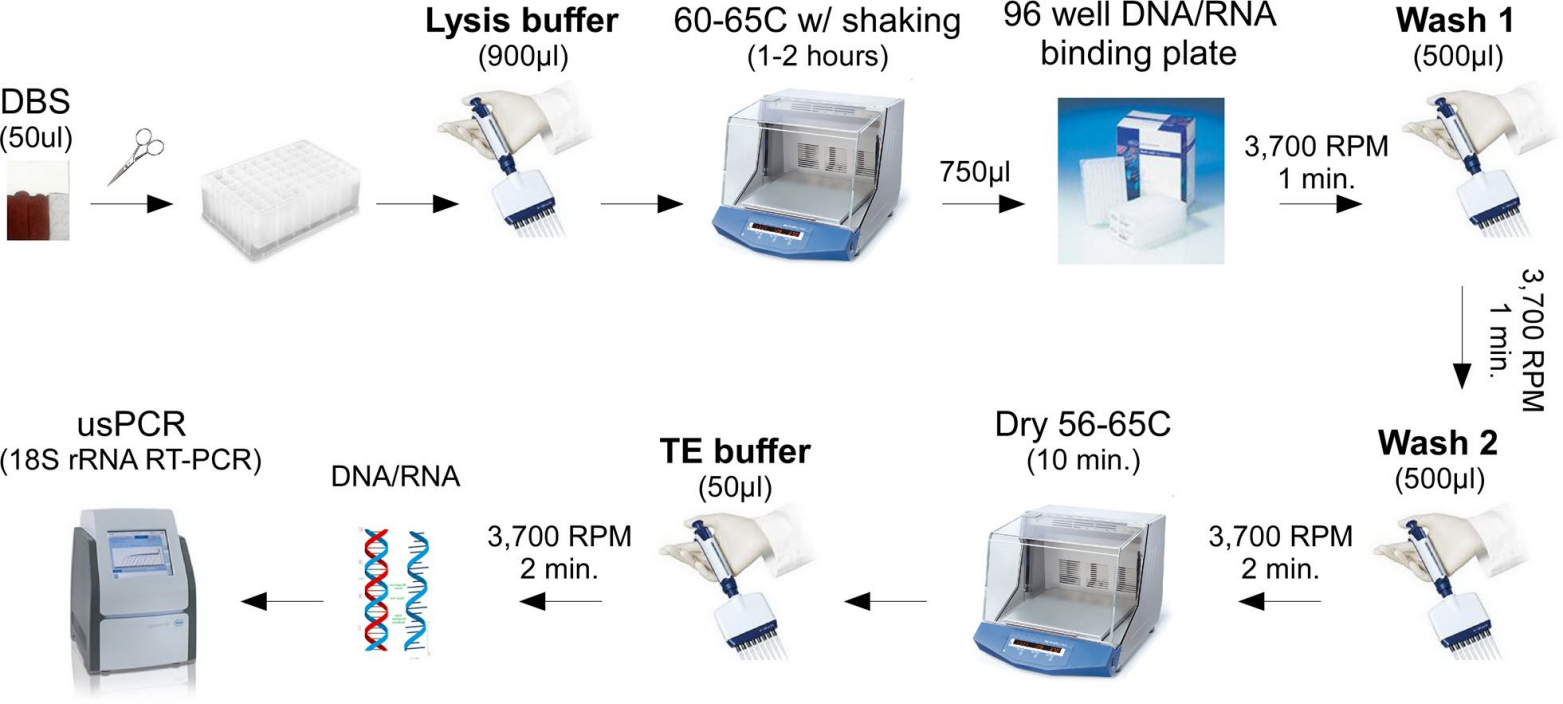
Schematic representation of the new extraction method. A detailed protocol can be found in Additional file 1

An ultrasensitive assay that uses capillary blood for the detection of *P. falciparum* and *P. vivax* has previously been published [21]. This method relies on reverse transcription PCR (RT-PCR) targeting the parasite 18S rRNA which exists in the thousands of copies per parasite and thus dramatically improves sensitivity (≤16 parasites/mL) [21, 24]. To adapt this ultrasensitive protocol to work on dried blood spots (DBS), a new extraction method was developed based on traditional guanidine and silica purification strategies. A multitude of different extraction conditions were empirically tested to determine the effect on purification of 18S rRNA from DBS (as assessed by quantitative RT-PCR for the *P. falciparum* 18S rRNA target using Qiagen QuantiTect multiplex RTPCR mastermix—with RT-mix for reverse transcription—and a Roche LC96 instrument [21]). This allowed for identification of conditions that incrementally improved extraction efficiency. Once an optimized method was found, effort was made to simplify the protocol by reducing the overall number and complexity of each step without compromising sensitivity. As a result, the final protocol was optimized both for sensitivity and simplicity (Fig. 1; Additional file 1).

Solutions were made in large batches to maximize efficiency and minimize variability. Lysis/Wash 1 solutions were made 20 L at a time and Wash 2 was made 10 L at a time, enough for at least 10,000 extractions. After every batch of newly made solutions, a control extraction was performed with previously created DBS samples of known parasitaemia, as a quality control measure to ensure batch-to-batch consistency. Solutions were aliquoted into 500 mL bottles and stored at room temperature (in the dark) for 2–3 months, or at 4 °C if for longer periods (detailed instructions in Additional files 2, 3). 2-mercaptoethanol (Sigma-Aldrich, St. Louis, MO, USA) was added fresh to each 500 mL bottle of Lysis solution (final concentration 0.5% v/v) before use.

### Testing of commercial buffer substitutes

Commercially available buffers tested in this study can found in Additional file 4. Commercial Lysis, Wash 1, and Wash 2 buffers that gave similar results as home-made new extraction method solutions were chosen for further testing in combination with each other until equal sensitivity as home-made buffers was achieved.

### Limit of detection for *Plasmodium falciparum*

Limit of detection (LoD) was established only for *P. falciparum*, because cultured *P. vivax* parasites are not available. Experiments using serial dilutions of unlysed whole blood mixed with cultured parasites with known densities were performed as previously reported [21] with the exception that dried blood spots were dried and stored in simulated field conditions (28 °C with 80% relative humidity for 2 weeks) before use in experiments. Data obtained from three independently created *P. falciparum* standards was used to obtain the LoD using a probit analysis in SAS version 9.2 (SAS Institute, Cary, NC, USA) [21].

### Field validation in Myanmar

**Fig. 2:**
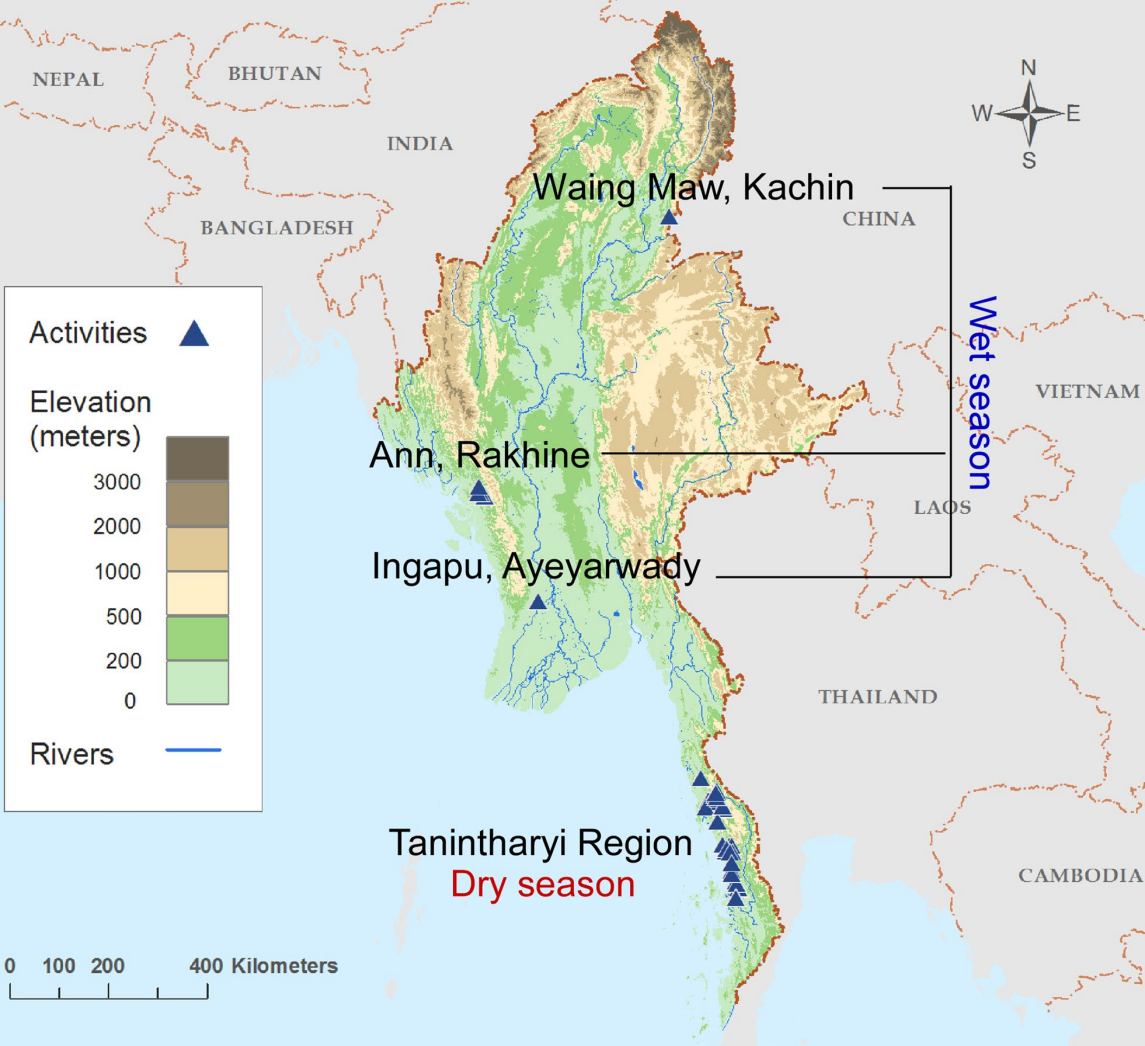
Map indicating sites in Myanmar where samples were collected during the wet and dry season

This study was part of a large multicenter cross-sectional malaria surveillance study conducted in 2015 in regions of Myanmar (Fig. 2). Samples were collected from regions with known high (Tanintharyi Region and Ann township, Rakhine State), moderate (Ingapu township, Ayeyarwady Region), and low (Waing Maw township, Kachin State) malaria case load, relative to GMS-level malaria burdens. Samples were collected in the dry season (March, 2015) from the Tanintharyi site, and wet season (June–September, 2015) from the remainder of sites.

**Fig. 3:**
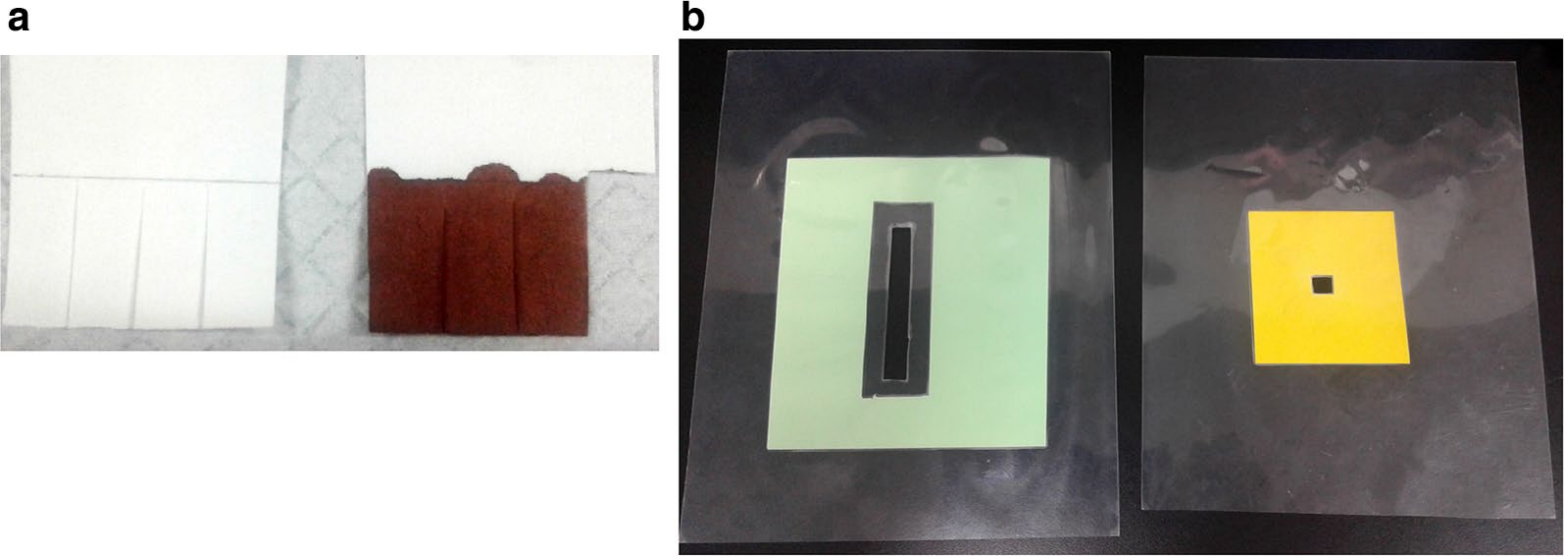
Sample collection and contamination minimization strategies. **a** Whatman 3MM filter paper is pre-cut to yield four 2 cm × 0.5 cm strips, each which corresponds to a 50 µL blood spot. Four horizontal cuts across a strip yield small enough pieces to fit into a standard 96 well plate (which has already been performed for the missing strip on the right), thereby reducing cutting time. **b** Plastic plate covers used to prevent cross-contamination. On the left is a typical plastic plate cover used during extraction and PCR setup which allows multichannel pipetting of an entire column (eight wells) but covers the remainder of the 96 well plate. The cover on the right is for a single well of a 96 well plate and is used during cutting to isolate the well receiving the cut DBS

Detailed field procedures have been described elsewhere [21]. From each volunteer, finger prick blood was collected into: (1) a capillary blood collection tube (0.3 mL); and (2) filter paper for dried blood spot (DBS) (50 µL). Capillary blood was stored in 0.75 mL DNA/RNA Shield preservative (Zymo Research, Irvine, CA, USA) to stabilize nucleic acids. For DBS, finger prick blood was collected using Whatman 3MM filter paper (pre-cut with four small tabs, each corresponding to a 2 cm × 0.5 cm area, which approximates a 50 µL blood volume) (Fig. 3a), and Whatman 903 Protein Saver (one circle, when available). DBS samples were air-dried, which varied from about 2 h during the dry season and up to 8 h during the wet season. After drying, samples were placed in a sealed plastic pouch with desiccant and stored at room temperature (7–10 months) until analyzed in the laboratory. The cross-sectional prevalence (the number of positive individuals per the number of individuals tested per site) of *P. falciparum* and *P. vivax* was determined using the 0.3 mL preserved capillary blood with Qiagen QIAamp DNA extraction method, and Whatman 3MM filter paper and 903 Protein Saver card with the new extraction method. The same 18S rRNA RT-PCR detection method was used for all samples [21].

### Statistical analysis

The kappa statistic (κ) is computed using the values generated in an N × N table (outcomes for each sample as negative, positive for *P. falciparum, P. vivax* or mixed), resulted from analyses by standard Qiagen extraction versus new extraction test. Assuming a normal distribution of data from a large pool of samples, the kappa confidence intervals were estimated using Spearman correlation statistics (with Fisher z transformation), using SAS software (version 9.2, SAS Institute, NC, USA). The κ > 0.75 indicates excellent agreement, 0.40 ≤ κ ≤ 0.75 represents fair or good agreement, and κ < 0.40 represents poor agreement.

### Assessment of the effect fungal growth on DBS on test sensitivity

Upon receipt by the laboratory, each DBS sample was visually inspected for apparent fungal contamination and the degree of contamination rated as none, mild, moderate, or severe. To determine whether a visible fungus growth affected the sensitivity of the RT-PCR assay, DBS samples with fungal contamination were independently tested, along with the corresponding preserved capillary blood (serving as the positive control).

### Minimization of contamination

Control of contamination in the lab started from the time of cutting DBS. It took approximately 2 h to cut DBS and prepare a 96-well plate in the lab (Additional file 5). The cutting time was significantly reduced by pre-cutting the Whatman 3MM filter paper (Fig. 3a). To minimize the chance of carry-over cross-contamination, plate covers were used during cutting, extraction and PCR procedures (Fig. 3b; Additional file 5). As an internal control, every eighth sample consisted of a blank filter paper sample that was cut, extracted and used for detection alongside actual field samples. This allowed for identification of contamination resulting from any step in the process that would otherwise yield false positives.

### Ethical consideration

The study was conducted as part of large-scale malaria surveillance study in Myanmar. The study protocol was independently reviewed and approved by the Institutional Review Boards of the University of Maryland School of Medicine, and the Ethics Review Committee of the Department of Medical Research, Myanmar Ministry of Health. All study participants provided written informed consent or assent with adult guardian consent as appropriate, and all study procedures were performed in accordance with the Declaration of Helsinki.

## Results

### A new extraction method for dried blood spots

A new extraction method for purification of nucleic acids from dried blood spots (DBS) was developed based on classic guanidine/silica methods. This method was refined by empirically testing how a number of different extraction variables impacted purification efficiency of *P. falciparum* 18S rRNA as determined by quantitative reverse-transcription polymerase chain reaction (qRT-PCR) (Additional file 6). A few key variables that improved sensitivity were: (1) decreasing the pH of the lysis buffer to slightly acidic conditions (pH 6.0–6.5); (2) including isopropanol at final concentration of 16.7% (v/v); (3) reconstituting the DBS with lysis buffer in the presence of heat and shaking (60–65 °C, 250 RPM, 1–2 h); (4) drying the DNA plate with heat prior to elution; and (5) using a higher volume of DBS (50 µL) (Additional files 6, 7). Once the desired sensitivity was achieved, the working protocol was simplified by reducing the number of steps without compromising sensitivity, to reduce the overall complexity and cost of the final protocol **(**Fig. 1; Additional file 1).

### Limit of detection for *Plasmodium falciparum*

When this finalized protocol was tested on laboratory created *P. falciparum* DBS standards (dried and stored in simulated field conditions of 28 °C, 80% relative humidity for 2 weeks) a limit of detection of 20 parasites/mL (or 1 parasite/50 µL DBS) for Whatman 3MM filter paper was obtained (Table 1). This is comparable to previously published ultrasensitive techniques using 2 mL venous blood or 0.3 mL preserved capillary blood (22 and ≤16 parasites/mL, respectively) [21, 22]. Using Whatman 903 Protein Saver cards, which are coated with a proprietary preservative (and more expensive), did not further improve sensitivity (23 parasites/mL).

**Table 1:** Limits of detection (LoD) for home-made and commercial new extraction method (NEM) buffers using Whatman 3MM dried blood spots

Step	Home-made	Commercial
Lysis	3M Guanidine thiocyanate 16.7% Isopropanol 2% Triton X100 10 mM EDTA 5 mM Trizma HCl pH 7.4 0.1% 6 N HCl 0.5% 2-mercaptoethanol pH 6.0–6.5	Qiagen RLT-plus 16.7% Isopropanol 0.5% 2-mercaptoethanol
Wash 1	Same as lysis but no 2-mercaptoethanol	Same as lysis but no 2-mercaptoethanol
Wash 2	25% Ethanol 25% Isopropanol 100 mM Sodium chloride 10 mM Trizma HCl pH 7.4	70% Ethanol 30% PBS
LoD	20 parasites/mL	22 parasites/mL

Since home-made solutions might not be possible in all settings, particularly in the field, commercially available buffers that could serve as suitable substitutes were tested (listed in Additional file 4). Qiagen RLT-plus with the addition of 16.7% isopropanol and 0.5% 2-mercaptoethanol was found to achieve a similar sensitivity (22 parasites/mL) as the home-made lysis buffer (Table 1; Additional file 8).

### Field validation in Myanmar

To confirm the utility of DBS for routine molecular surveillance of asymptomatic malaria, two different field trials in Myanmar were undertaken (Fig. 2). The cross-sectional prevalence of *P. falciparum* and *P. vivax* was determined using either the current DBS-based method or a previously described ultrasensitive method utilizing 0.3 mL preserved capillary blood (detection in both cases was with 18S rRNA RT-PCR [21]). Table 2 shows that both methods yielded near-identical prevalence rates for both *P. falciparum* and *P. vivax* malaria in the wet season, from a total of 593 asymptomatic individuals living in Rakhine State, Kachin State and Ayeyarwady Region. For the dry season survey of 1739 asymptomatic participants in Tanintharyi Region, the prevalence of *P. falciparum* was near-identical between the two methods, while *P. vivax* was slightly lower by DBS (5.3% by DBS versus 5.9% by capillary blood), though this difference was not statistically significant (Table 3).

**Table 2:** Malaria prevalence rates during the wet season from three sites in Myanmar comparing preserved capillary blood to dried blood spots (DBS)

Species	Rakhine (n = 198)		Ayeyarwady (n = 195)		Kachin (n = 200)		Overall (n = 593)		
	Blood/Qiagen	DBS/NEM	Blood/Qiagen	DBS/NEM	Blood/Qiagen	DBS/NEM	Blood/Qiagen	DBS/NEM	Kappa (95% CI)
*Pf* (%)	11.0	11.0	7.7	8.7	0.5	0.5	6.4	6.7	0.92 (0.85–0.98)
*Pv* (%)	8.5	8.5	5.1	5.6	1.0	0.0	4.9	4.7	0.89 (0.80–0.98)
Mixed (%)	3.5	3.5	0.0	0.0	0.0	0.0	1.2	1.2	1.0 (1.0–1.0)

Pf, *Plasmodium falciparum* monoinfection; Pv, *P. vivax* monoinfection; Mixed, Mixed Pf and Pv; Blood, preserved capillary blood; Qiagen, Qiagen extraction method; DBS, Whatman 3MM dried blood spot; NEM, new extraction method; 95% CI., 95% confidence interval

**Table 3:** Malaria prevalence rates during the dry season from the Tanintharyi Region of Myanmar comparing preserved capillary blood to dried blood spots (DBS), using Qiagen extraction method and new extraction method (NEM), respectively (n = 1739)

Species	Blood/Qiagen	DBS/NEM	Kappa (95% CI)
*Pf* (%)	1.1	1.2	0.85 (0.73–0.97)
*Pv* (%)	5.9	5.3	0.86 (0.81–0.92)
Mixed (%)	0.3	0.1	0.57 (0.13–1.0)

Pf, *Plasmodium falciparum* monoinfection; Pv, *P. vivax* monoinfection; Mixed, Mixed Pf and Pv; DBS, Whatman 3MM dried blood spot; NEM, new extraction method; 95% CI, 95% confidence interval

### Minimizing contamination

Increased sensitivity is oftentimes accompanied by decreased specificity. The use of plate covers and strict contamination-preventing protocols are critical for minimizing cross-contamination events (Fig. 3b) [21]. Dried blood spots however have an added risk where cutting itself can present an additional source of contamination. Previous work has attempted to resolve this problem through the incorporation of laser-cutting technology, and while effective it is not readily field deployable [25].

**Fig. 4:**
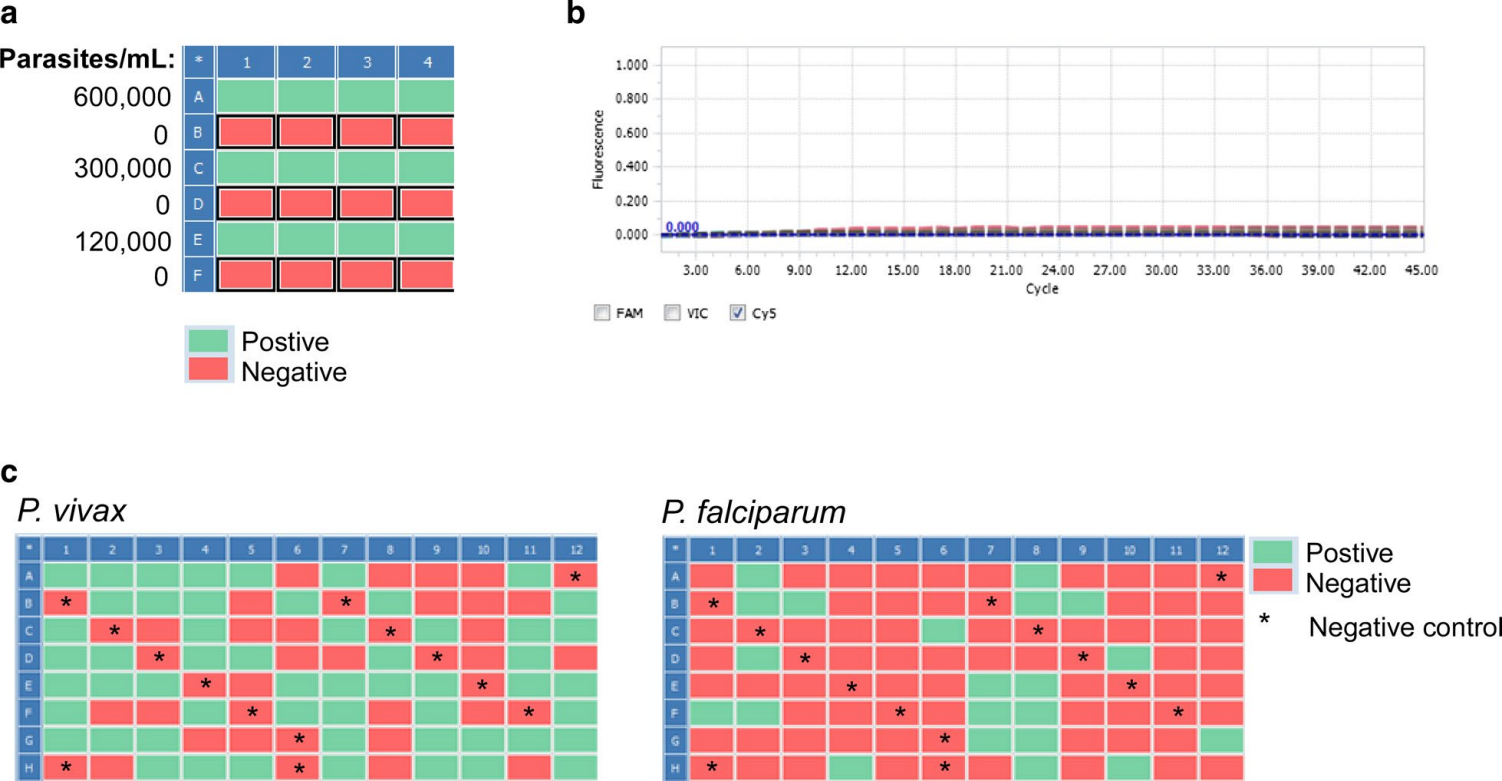
Controlling for contamination and false positives. **a** Wiping scissors and forceps three times with a kimwipe sprayed with 70% ethanol is sufficient to prevent contamination when cutting from a high to a zero parasitemic sample. **b** The amplification curves for all of the 0 parasites/mL samples are shown indicating lack of amplification. **c** Representative plates from a field survey from Myanmar showing that even in high malaria burden areas, negative control samples consisting of blank filter papers (indicated by asterisk) remain negative

As a result, control experiments were undertaken to determine the minimal safeguards needed to reliably prevent contamination from cutting itself. Three wipes of the scissor blades and forceps with a damp paper towel (or kimwipe) sprayed with 70% ethanol was found to be sufficient to consistently prevent contamination when cutting from a high (600,000 parasites/mL) to a zero parasitemic sample (Fig. 4a, b; Additional file 5). These results bore out in field surveys where the incorporation of these safeguards was sufficient to prevent the appearance of false-positives in blank filter paper negative controls, even in high malaria burden areas (Fig. 4c).

### Fungal growth

Finally, the collection of dried blood spots during the rainy season (and associated high humidity) can result in the appearance of fungal contamination. The quality of these samples is unknown and so they are often discarded. To determine how the presence of fungus on DBS affected the sensitivity of the current assay, results obtained from filter paper samples containing fungal contamination were compared to those obtained from matched preserved capillary blood samples (which served as a positive control).

In contrast to Whatman 3MM, paired dried blood spots collected on 903 Protein Saver cards were found to have no visible evidence of fungal contamination in any instance. 903 Protein Saver cards, therefore, can be used to prevent fungal growth for sample collection during the rainy season (and in this instance it also served as a useful non-fungal DBS control). Table 4 shows a comparison of the qualitative (positive or negative) results from 3MM DBS samples with mild, moderate or severe fungal growth, paired with the corresponding preserved capillary blood and 903 Protein Saver cards. As can be seen, the presence of fungus had no qualitative impact on the assay’s ability to identify *P. falciparum* or *P. vivax* parasites in the majority (14 of 15) of fungus-contaminated samples, even when fungal contamination was rated as severe (Table 4). Although the sample size was small, there also surprisingly was no quantitative loss in qPCR cycle threshold (Ct) values in malaria positive samples that had fungus compared to those that did not (Additional file 9).

**Table 4:** The presence of fungus has minimal effect on the detection of *P. falciparum* and *P. vivax* from Whatman 3MM dried blood spots (DBS) compared to paired preserved capillary blood and Whatman 903 Protein Saver cards

Sample#	Blood	903 DBS		3MM DBS	
	Malaria	Malaria	Fungus	Malaria	Fungus
1	Pf+	Pf+	None	Pf+	Slight
2	Pv+, Pf+	Pv+, Pf+	None	Pv+, Pf+	Slight
3	Pv+	Pv+	None	Pv+	Slight
4	Pv+	Pv+	None	Pv+	Slight
5	Pv+	Pv+	None	Negative	Slight
6	Pf+	Pf+	None	Pf+	Slight
7	Pv+	Pv+	None	Pv+	Slight
8	Pf+	Pf+	None	Pf+	Moderate
9	Pv+	Pv+	None	Pv+	Moderate
10	Pf+	Pf+	None	Pf+	Moderate
11	Pv+	Pv+	None	Pv+	Moderate
12	Pv+, Pf+	Pv+, Pf+	None	Pv+, Pf+	Moderate
13	Pv+	Pv+	None	Pv+	Severe
14	Pv+, Pf+	Pv+, Pf+	None	Pv+, Pf+	Severe
15	Pv+	Pv+	None	Pv+	Severe

# Individual study participant; Pf, *P. falciparum*; Pv, *P. vivax*; +, positive; 903 DBS, Whatman 903 Protein Saver dried blood spot; 3MM DBS, Whatman 3MM dried blood spot

## Discussion

This report describes a new nucleic acid extraction method enabling the ultrasensitive PCR detection of *P. falciparum* and *P. vivax* from dried blood spots. This method is less expensive and more field-scalable than currently available protocols [21, 22] and can be used for large scale molecular surveillance studies in resource limited settings. This is the first report of a blood spot-based ultrasensitive method that has a comparable limit of detection as whole-blood based methods, capable of detecting the very low density parasitaemias associated with asymptomatic infections in low transmission settings.

Attempts have previously been made to improve the sensitivity of DBS samples for use in malaria surveillance studies [26–31]. The marked improvement in sensitivity of this assay was achieved in two ways: (1) by developing a new extraction method that improved nucleic acid purification from DBS; and (2) taking advantage of the amplification of 18S rRNA, which exists in the thousands of copies per parasite [24]. This high copy number, coupled with the exquisite chemical stability of 18S rRNA, resulted in the sensitivity of 1 parasite per 50 µL DBS that was achieved. While this is below what would be expected based on a Poisson distribution of intact parasites, it is consistent with what others have reported from whole blood, likely reflecting the presence of circulating cell free parasite nucleic acids [32]. Although chemically stable, circulating parasite 18S rRNA has been reported in previous controlled human infections to be undetectable 5 days post-treatment in half of participants (and reduced by >2 log_10_ parasites/mL in the other half); and undetectable in all participants at a subsequent 28 day follow-up [24]. This should reduce the worry of lingering parasite 18S rRNA leading to false-positives during epidemiological surveys.

Existing ultrasensitive detection methods incorporate the use of venous or capillary blood [21, 22]. Both achieve a remarkable sensitivity of 22 and ≤16 parasites/mL, respectively, a sensitivity many 1000-folds higher than what is achieved with standard malaria diagnostic methods. However, the utility of these methods is limited by their need for special collection, handling, and storage conditions that are not always attainable in resource-limited, malaria endemic settings.

Since the current method removes many of these hindrances, it should help enable easier routine molecular surveillance and other research studies of large populations, which are frequently needed for malaria surveillance and epidemiologic/clinical research. It may also prove useful in understanding the true burden of pregnancy-associated malaria, where peripheral parasitaemias are often quite low due to sequestration in the placenta. An added benefit is that both collection and extraction of DBS with this new method are substantially less expensive than current methods. Based on list prices, the approximate cost of the home-made and commercial protocols, including all consumables needed for extraction, is $1.77 and $2.66 per sample, respectively, whereas Qiagen QIAamp is $7.87 per sample. This results in savings of almost $100,000 in extraction costs alone for a survey of 15,000 samples (the approximate number of samples tested using this method in a recent national malaria survey in Myanmar).

It should be noted that while this technique is optimized for extraction of RNA, it also co-purifies parasite DNA. This has certain advantages, allowing for downstream genomic analyses such as PCR amplification and DNA sequencing of parasite drug resistance markers. In fact, the same eluates currently used for ultrasensitive RT-PCR are also suitable for *kelch13* (K13) sequencing. Preliminary experiments have shown that performing the longer 2-h incubation with lysis buffer at 60–65 °C increased the success rate of K13 sequencing with no effect on the LoD for usPCR. If no downstream genomic applications are required, the shorter 1-h incubation is sufficient.

This technology was recently transferred to the Department of Medical Research (DMR), Myanmar Ministry of Health and Sports, in Yangon, Myanmar, where local scientists using this method have screened more than 25,000 dried blood spots for *P. falciparum* and *P. vivax* in the past year. This includes the recent U.S. President’s Malaria Initiative-sponsored national Malaria Indicator Survey, which consisted of more than 14,000 DBS and represents the largest survey done in the history of Myanmar’s malaria work (the results of this survey will be reported elsewhere). The reduced cost and procedural simplicity in both sample collection and extraction made this large national-level molecular surveillance study feasible. And while this test is not a point-of-contact test, its high-throughput nature makes it ideal for screening such large numbers of samples. Highlighting this, the current throughput at DMR is up to 720 samples per day, allowing for quick turn-around of even the largest surveys to inform malaria public health strategies in a timely fashion. Additionally, as DBS are considered non-regulated and non-biohazardous agents, this new method allows for easier shipping and transportation of samples to central laboratories, whether located in-country or abroad.

This method can also be applied retrospectively to archival DBS material to obtain a better understanding of historic malaria trends. As the DBS used in the field validation studies had been stored for up to 10 months at ambient conditions in Myanmar, it appears storage for at least this long has minimal effect on sensitivity. Consistent with this observation, laboratory-created samples stored in ambient conditions in Myanmar for 3 months showed no quantitative loss in 18S rRNA qPCR cycle threshold (Ct) values as compared to samples analyzed immediately after creation (Additional file 10). Previous reports examining the effects of longer-term DBS storage (though not 18S rRNA *per se*) have revealed two important findings: (1) storage of DBS at −20 °C appears to almost completely protect against nucleic acid degradation [33, 34]; (2) prolonged storage at room temperature impairs the ability to amplify large amplicons, but appears to minimally affect smaller amplicons, even after 10 years of storage [35–37]. It should therefore be noted that the 18S amplicons used in this RT-PCR assay for *P. falciparum* and *P. vivax* are quite small (104 and 106 bp, respectively) thereby helping minimize negative effects from nucleic acid degradation from prolonged storage. Further, the RT-PCR method incorporates a human actin internal control, allowing for a quantitative assessment of sample integrity based on relative actin Ct value differences (Additional file 11). It is important to note that actin values are based on white blood counts and thus will vary based on sex, age and other parameters during field trials (Additional file 12). Thus, only dramatic changes (or complete loss in signal) for actin, or relative changes within the same sample set, are helpful in pinpointing potential problems.

Finally, the improved ability to extract nucleic acids from DBS may prove useful for other blood-borne infections, particularly those resulting from other protozoans. Targeting these organisms’ respective ribosomal RNA would be a relatively straightforward adaptation of the current RT-PCR method. Bacterial, viral or other blood-borne pathogens may also be prime targets, though the stability of each respective molecular target and PCR strategy should be verified a priori. And finally, as whole genome and transcriptome sequencing technologies improve, it is possible that DBS will serve as a useful starting material for the better understanding and characterization of both communicable and non-communicable diseases as well.

## Conclusions

The ability to use dried blood spot samples for ultrasensitive detection of malaria will facilitate molecular surveillance studies to understand the role of asymptomatic infections in malaria pathogenesis, emergence and spread of drug resistance, and malaria transmission. These tests can be performed in public health laboratories in malaria-endemic countries, where they are already being used to target elimination interventions such as mass drug administration. While basic laboratory infrastructure is still required for this assay, it is dramatically simpler and less expensive than existing methods, in terms of both sample collection, transport, and processing. Additionally, the recent experience in Myanmar demonstrates that tens of thousands of samples can be readily analyzed in-country by local staff using this method. While such capable laboratories may not exist in every malaria-endemic country, the use of blood spots permits transport of samples to locations where central laboratories do exist. Further evaluations are needed to assess how this DBS-based ultrasensitive method can be coupled with loop-mediated isothermal [38] technologies to bring it closer to a highly sensitive point-of-contact test for malaria, with the potential to be adapted for detecting other diseases.

## Additional files

**Additional file 1.** A detailed working protocol for performing the new extraction method.

**Additional file 2.** Step by step instructions for making large batches of new extraction method solutions.

**Additional file 3.** A comprehensive list of all consumables and equipment used in performing the new extraction method.

**Additional file 4.** A list of commercial lysis and wash buffers tested in comparison to home-made new extraction method buffers.

**Additional file 5.** A video demonstrating proper cutting technique for preventing cross-contamination.

**Additional file 6.** A schematic representing the different extraction variables that were tested (represented by text in the arrows) and the resulting refinements that were made to the new extraction method. Results from these experiments can be found in Additional files 7A–F.

**Additional file 7.** Testing a number of extraction variables to improve Ct value. Tested variables include different sources of guanidine thiocyanate (7A), lysis buffer conditions (7B), temperature and incubation time (7C), the size of DBS used (7D), wash conditions (7E), and elution conditions (7F).

**Additional file 8.** Testing of commercial buffers in comparison to home-made buffers on the purification efficiency of *Plasmodium falciparum* 18S rRNA from dried blood spots as assessed with a reverse-transcription PCR assay. Each table represents an independent experiment using DBS samples with varying parasitaemias (400 – 2,000 parasites/mL). A lower cycle threshold (Ct) value indicated improved efficiency. More information about the various buffers can be found in Additional file 4. GuSCN, guanidine thiocyanate; 2Me, 2-mercaptoethanol; ISOH, isopropanol; ETOH, ethanol; PBS, phosphate buffered saline; SD, standard deviation.

**Additional file 9.** The growth of fungus has no effect on cycle threshold (Ct) values for detection of *Plasmodium falciparum* or *P. vivax* from dried blood spots as assessed with a reverse-transcription PCR assay for 18S rRNA. SD, standard deviation.

**Additional file 10.** *Plasmodium falciparum* dried blood spot samples (400 parasites/mL) stored at room temperature in Myanmar for 3 months show similar cycle threshold (Ct) values as samples created and analysed immediately using a reverse-transcription PCR assay for *P. falciparum* 18S rRNA. Samples were created contemporaneously, with the only difference in storage conditions. PS, protein saver; RT, room temperature; SD, standard deviation.

**Additional file 11.** Representative cycle threshold (Ct) values for *Plasmodium falciparum* 18S rRNA and human actin RNA from dried blood spot standards (n=4) as assessed with a reverse-transcription PCR assay. SD, standard deviation.

**Additional file 12.** The range, median, mean and standard deviation for human actin cycle threshold (Ct) values from a field survey in Myanmar (n=165) as assessed with a reverse-transcription PCR assay. SD, standard deviation.
