# Supplementary material for: A novel method for extracting nucleic acids from dried blood spots for ultrasensitive detection of low-density Plasmodium falciparum and Plasmodium vivax infections
Source: Malar J. 2017 Sep 18;16:377. doi: 10.1186/s12936-017-2025-3 (PMC5604154; doi:10.1186/s12936-017-2025-3)
Supplement: Supplementary file 1 — Additional file 1. A detailed working protocol for performing the new extraction method. [file 12936_2017_2025_MOESM1_ESM.doc]

**Filter Paper New Extraction Method (NEM) Working Protocol**

**CHECK EACH STEP OF PROTOCOL AFTER COMPLETING:**

**Equipment needed:**

**Materials needed *:**

1. Plate covers
2. Scissors
3. Forceps
4. Plate centrifuge
5. Shaking oven incubator
6. 1mL multichannel pipette (and tips)
7. 200µL multichannel pipette (and tips)
8. Spray bottle (70% ethanol)
9. Film sealing roller
10. Lysis, Wash 1, Wash 2 (see table below)
11. TE (Tris-EDTA) buffer (pH 8.0)
12. Omega E-Z 96-Well DNA Filter Plate**
13. Pre-labeled 96 deep well plate, 2.2ml (cutting)
14. 96 deep well collection plate, 2ml
15. 96 well elution plate, 0.5ml
16. Kimwipes
17. 96 well aluminum sealing foils
18. Reservoirs (for multichannel use)

|  | **Homemade**  **NEM Buffers*** | **Commercial**  **NEM Buffers** |
| --- | --- | --- |
| **Lysis:** | 3M Guanidine thiocyanate**@**  16.7% Isopropanol  2% Triton X100  10mM EDTA  5mM Trizma HCl pH 7.4  0.1% 6N HCl  0.5% 2-mercaptoethanol  pH 6.0-6.5 | Qiagen RLT-plus@  16.7% Isopropanol  0.5% 2-mercaptoethanol |
| **Wash 1:** | Same as lysis but no  2-mercaptoethanol | Same as lysis but no  2-mercaptoethanol |
| **Wash 2:** | 25% Ethanol  25% Isopropanol  100mM Sodium Chloride  10mM Trizma HCl pH 7.4 | 70% ethanol, 30% PBS |

**BEFORE STARTING:**

A) Turn on oven incubator (60-65C)

B) Clean covers (96 well covers)

C) Make sure lysis buffer contains 2-mercaptoethanol (2.5ml for 500ml Lysis Solution)

* A detailed protocol for large-batch creation of homemade NEM buffers can be found in Additional file 2. All required items, including catalogue numbers, can be found in Additional file 3.

** If Omega E-Z plates cannot be obtained, DNA plates from QiaAMP 96 kits can be used as suitable replacement. The original protocol used ACROPREP 1ml DNA Binding plates but due to a manufacturing change these are no longer suitable for this protocol.

**NOTE: Lysis and Wash 1 contain guanidine thiocyanate which is INCOMPATIBLE with bleach**

**PROTOCOL:**

1) Cut 50µL DBS into small enough pieces to place inside a labeled 96 well deep well plate

- See Additional file 4 or <https://www.youtube.com/watch?v=K129KmLh_Ok&t=1s>

- Wipe scissor blades and forceps 3 times with Kimwipe sprayed with 70% ethanol

- Use plate cover

2) With 1ml multichannel pipette add 900ul of **Lysis Buffer** to each well

- Use plate covers

-Make sure Lysis Buffer contains 0.5% 2-mercaptoethanol

3) Check to make sure all DBS samples are submerged in the Lysis Buffer

- Seal with aluminum foil forcefully and quick spin the plate

4) Incubate at 60-65C (with shaking 250RPM) for **1-2 hours**

- Do not secure plates in incubator by placing tape on the top of the plate, place on the sides

- If downstream genomic applications are required, incubate for 2 hours; otherwise 1 hour is

sufficient for usPCR

5) While waiting, place the 96-Well DNA Filter Plate on top of a 96 deep well collection plate (2ml)

- Put a piece of tape on each side to connect the DNA plate to the 96-well deep well plate

6) After 60-65C incubation is complete, spin down plates briefly (3,700RPM for a few seconds) to collect samples to the bottom

7) Transfer 750µl from this plate (Lysis Buffer) to the DNA Plate

- Use plate covers for this and all remaining steps

8) Spin 3,700RPM for 1min

- After spin, check to see if any liquid left in wells (usually no); if yes spin again for 5min

9) Wash with 500ul **Wash Buffer 1** (use the same 96 deep well collection plate)

- Spin 3,700RPM for 1min

10) Wash with 500ul **Wash Buffer 2** (use the same 96 deep well collection plate)

- Spin 3,700RPM for 2min

11) Place DNA plate on top of 0.5ml 96 well elution plate and dry in 56-65C incubator for 10min

- Do not cover, no shaking

12) With 200µl multichannel add 50uL of **TE buffer** directly to center of DNA plate wells to elute

- Spin down 3,700 RPM for 2 min

13) Label/seal and store at -80C
