# Supplementary material for: A novel method for extracting nucleic acids from dried blood spots for ultrasensitive detection of low-density Plasmodium falciparum and Plasmodium vivax infections
Source: Malar J. 2017 Sep 18;16:377. doi: 10.1186/s12936-017-2025-3 (PMC5604154; doi:10.1186/s12936-017-2025-3)
Supplement: Supplementary file 2 — Additional file 2. Step by step instructions for making large batches of new extraction method solutions. [file 12936_2017_2025_MOESM2_ESM.doc]

**LARGE BATCH PREPARATION OF NEM SOLUTIONS**

**This protocol is for making New Extraction Method (NEM) solutions for use with dried blood spots (DBS). This will make enough solutions for at least 10,000 samples.**

**CHECK EACH STEP OF PROTOCOL AFTER COMPLETING:**

**Materials needed *:**

1. Molecular grade H2O
2. 10ml serological pipette
3. Pre-labeled 500ml bottles**
4. 20-25L Carboy (with spigot)
5. 5-10L Carboy (with spigot)
6. 2-mercaptoethanol
7. Large funnel
8. pH meter
9. 100% Ethanol
10. 100% Isopropanol
11. Guanidine Thiocyanate
12. Triton X100
13. 0.5M EDTA Solution (pH 8)
14. 1M Trizma Hydrochloride solution (pH 7.4)
15. 6N/6M Hydrochloric acid
16. 5M Sodium chloride solution

* All items, including catalogue numbers, can be found in Additional file 3

** For aliquoting Lysis, Wash 1 and Wash 2 solutions; if purchasing molecular grade water, the resulting empty bottles can be used instead

**NOTE: Guanidine thiocyanate containing solutions are INCOMPATIBLE with bleach**

**1) Lysis/Wash 1 buffer (*20L*):**

1. To a graduated 20-25L Carboy add **5L** of molecular biology grade H2O
   1. To speed dissolving of chemicals, water can be preheated to 50-60C
2. Place a large funnel on the carboy & add **7 Kg** of Guanidine thiocyanate: **7 (1kg bottles)**
   1. No need to weigh, just add entire contents of bottles
3. Add Triton X100: **400ml**
4. Add 0.5M EDTA: **400ml**
5. Add 1M Trizma hydrochloride ph 7.4: **100ml**
6. Add Isopropanol (2-propanol): **3,340ml** = **6 (500ml bottles) + measure 340ml**
7. Add 6N or 6M HCl: **20ml** *(use pipetteman and 10ml pip*ette)
   1. Be *VERY* careful, this is a very strong acid (wear gloves, labcoat, goggles!)
8. Bring up to 20L with molecular biology grade water
9. Shake vigorously every 15 minutes until dissolved (or let sit overnight)
10. Check to make sure chemicals are dissolved
11. Once dissolved, double-check that solution is at 20L mark, if not add more water **and mix**
12. Take a small aliquot and check pH (should read between 6.3-6.5)
    1. It is important the pH is below 6.5, if pH is not within 6.3-6.5 this is an indication something may have gone wrong as the protocol should yield a pH within this range
13. Aliquot 500ml into pre-labeled bottles: **26 Lysis bottles*** and **14 Wash 1 bottles**
14. Store at 4°C (preferably in the dark) long-term, or room temperature for few months

* Make sure to add 2.5ml of 2-mercaptoethanol before use

**2) Wash buffer 2 (*10L*)**

To a graduated 10L Carboy add:

1) **2.5L** of 100% Ethanol: **5 (500ml bottles)**

2) **2.5L** of 100% Isopropanol (2-propanol): **5 (500ml bottles)**

3) Take an empty 500mL H2O bottle and pour:

- - **100ml 1M Trizma hydrochloride ph 7.4**
  - **200ml 5M NaCl**
    - - - **200ml molecular biology H2O**
        - Pour contents into 10L container

5) **4.5L** of Molecular biology H2O**: 9 (500ml bottles)**

6) Mix and pour into **20 (500ml) Wash 2 bottles**
