# Supplementary material for: A novel method for extracting nucleic acids from dried blood spots for ultrasensitive detection of low-density Plasmodium falciparum and Plasmodium vivax infections
Source: Malar J. 2017 Sep 18;16:377. doi: 10.1186/s12936-017-2025-3 (PMC5604154; doi:10.1186/s12936-017-2025-3)
Supplement: Supplementary file 4 — Additional file 4. A list of commercial lysis and wash buffers tested in comparison to home-made new extraction method buffers. [file 12936_2017_2025_MOESM4_ESM.docx]

**Additional file 4. A list of commercial lysis and wash buffers tested in comparison to home-made new extraction method buffers.**

| **Lysis buffer** | **Vendor** |
| --- | --- |
| RLT Plus | Qiagen |
| RLT | Qiagen |
| AL | Qiagen |
| ATL | Qiagen |
| Purelink RNA lysis buffer | Thermo Fisher |
| Purelink Viral Lysis Buffer | Thermo Fisher |
| Genelute buffer | Sigma Aldrich |
| Guanidine thiocyanate solution 6M | Sigma Aldrich |
| Guanidine hydrochloride solution 6M | VWR |
| **Wash 1** |  |
| RW1 | Qiagen |
| AW1 | Qiagen |
| RLT Plus | Qiagen |
| RLT | Qiagen |
| Wash buffer 1 | Sigma |
| Purelink Wash 1 | Thermo Fisher |
| **Wash 2** |  |
| AW2 | Qiagen |
| RPE | Qiagen |
| RWT | Qiagen |
| Wash buffer 2 | Sigma |
| Purelink Wash 2 | Thermo Fisher |
| 70% ethanol | Sigma |
| 70% ethanol, 30% PBS | PBS, VWR |
