# Supplementary figures and images for: A novel method for extracting nucleic acids from dried blood spots for ultrasensitive detection of low-density Plasmodium falciparum and Plasmodium vivax infections
Source: Malar J. 2017 Sep 18;16:377. doi: 10.1186/s12936-017-2025-3 (PMC5604154; doi:10.1186/s12936-017-2025-3)

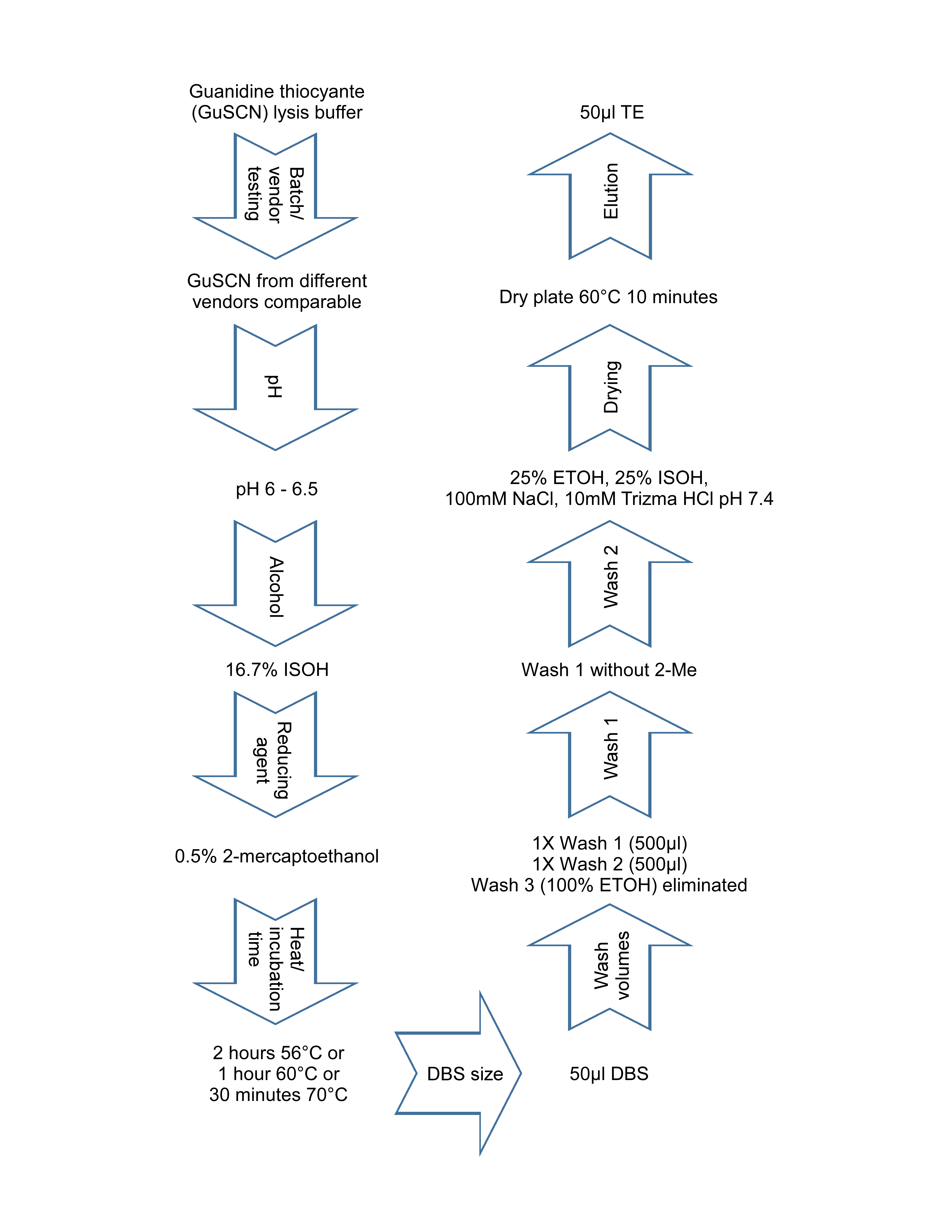

Supplement: Supplementary file 6 — Additional file 6. A schematic representing the different extraction variables that were tested (represented by text in the arrows) and the resulting refinements that were made to the new extraction method. Results from these experiments can be found in Additional files 7A–F. [file 12936_2017_2025_MOESM6_ESM.tif]
