# Supplementary material for: A novel method for extracting nucleic acids from dried blood spots for ultrasensitive detection of low-density Plasmodium falciparum and Plasmodium vivax infections
Source: Malar J. 2017 Sep 18;16:377. doi: 10.1186/s12936-017-2025-3 (PMC5604154; doi:10.1186/s12936-017-2025-3)
Supplement: Supplementary file 7 — Additional file 7. Testing a number of extraction variables to improve Ct value. Tested variables include different sources of guanidine thiocyanate (7A), lysis buffer conditions (7B), temperature and incubation time (7C), the size of DBS used (7D), wash conditions (7E), and elution conditions (7F). [file 12936_2017_2025_MOESM7_ESM.docx]

**Additional file 7.** **Testing a number of extraction variables to improve Ct value**

**7A.** **Different commercial sources of guanidine thiocyanate show similar extraction efficiency of *Plasmodium falciparum* 18S rRNA from dried blood spots as determined using a reverse-transcription PCR assay using dried blood spots.** Similar cycle threshold (Ct) values indicate comparable extraction efficiency from 100-200 parasite/mL DBS samples.

| Vendor: | Sigma | Amresco | RPI |
| --- | --- | --- | --- |
| Price/kg: | $623 | $363 | $386 |
| Average Ct  (± SD) | 29.4  (±.18) | 29.3  (±.41) | 29.4  (±.57) |

| Vendor: | RPI | Fisher |
| --- | --- | --- |
| Price/kg: | $386 | $409 |
| Average Ct  (± SD) | 28.4  (±.03) | 28.1  (±.24) |

**7B.** **The effect of different lysis buffer conditions on the extraction efficiency of *Plasmodium falciparum* 18S rRNA from dried blood spots as assessed by a reverse-transcription PCR assay.** Each table represents an independent experiment using DBS samples with varying parasitemias (10^1^ – 10^4^ parasites/mL). A lower cycle threshold (Ct) value indicates improved extraction efficiency. ISOH, isopropanol; ETOH, ethanol; 2-Me, 2-mercaptoethanol; TCEP, Tris (2-carboxyethyl) phosphine hydrochloride; SD, standard deviation.

| pH: | 7.5 | 6.5 | 5.5 |
| --- | --- | --- | --- |
| Average Ct  (± SD) | 22.6  (±.6) | 21.4  (±.2) | 21.4  (±.6) |

| Isopropanol: | 0% | 25% |
| --- | --- | --- |
| Average Ct  (± SD) | 37.8  (±1.2) | 27.5  (±.16) |

| Isopropanol: | 10% | 16.7% | 25% |
| --- | --- | --- | --- |
| Average Ct  (± SD) | 28.5  (±.3) | 21.6  (±.3) | 23.1  (±.3) |

| Alcohol: | 16.7% ISOH | 16.7% ETOH |
| --- | --- | --- |
| Average Ct  (± SD) | 28  (±.32) | 28.9  (±.37) |

| Reducing agent: | - | 2-Me (1%) | TCEP (25mM) |
| --- | --- | --- | --- |
| Average Ct  (± SD) | 31.2  (±.03) | 28.4  (±.18) | 28.7  (±.18) |

| 2-Me: | 0.1% | 0.33% | 1% |
| --- | --- | --- | --- |
| Average Ct  (± SD) | 24.9  (±.08) | 24.8  (±.10) | 25.0  (±.01) |

| Cellulase: | - | 100 Units |
| --- | --- | --- |
| Average Ct  (± SD) | 27.0  (±.69) | 27.0  (±.35) |

**7C.** **The effect of temperature and incubation time (with lysis buffer) on the extraction efficiency of *Plasmodium falciparum* 18S rRNA from dried blood spots as assessed by a reverse-transcription PCR assay.** Each table represents an independent experiment using DBS samples with varying parasitemias (200 – 4,000 parasites/mL). A lower cycle threshold (Ct) value indicates improved efficiency. RT, room temperature; O/N, overnight; min, minutes; SD, standard deviation.

| Temperature: | RT | | 56°C | |
| --- | --- | --- | --- | --- |
| Duration: | 15 min | O/N | 15 min | O/N |
| Average Ct  (± SD) | 27.2  (±.09) | 27.9  (±.06) | 25.5  (±.04) | 33.7  (±.43) |

| Temperature: | 56°C | | | |
| --- | --- | --- | --- | --- |
| Duration: | 30 min | 1 hour | 2 hours | 4 hours |
| Average Ct  (± SD) | 26.9  (±.04) | 24.9  (±.06) | 24.4  (±.10) | 24.6  (±.20) |

| Temperature: | 56°C | | 60°C | | 75°C |
| --- | --- | --- | --- | --- | --- |
| Duration: | 1 hour | 2 hours | 1 hour | 2 hours | 1 hour |
| Average Ct  (± SD) | 28.0  (±.13) | 27.2  (±.20) | 27.1  (±.09) | 27.2  (±.65) | 27.2  (±.23) |

| Analyte: | Whatman 3MM | | | Whatman 903 | | |
| --- | --- | --- | --- | --- | --- | --- |
| Temperature: | 56°C | 60°C | 70°C | 56°C | 60°C | 70°C |
| Duration: | 2 hour | 1 hour | 30 min | 2 hour | 1 hour | 30 min |
| Average Ct  (± SD) | 29.1  (±.63) | 29.2  (±.90) | 29.1  (±.28) | 28.9  (±.82) | 28.7  (±.23) | 28.5  (±.45) |

**7D.** **The effect of dried blood spot (DBS) size on the extraction and detection of *Plasmodium falciparum* 18S rRNA with a reverse-transcription PCR assay using a 80 parasite/mL sample.** Ct, cycle threshold; SD, standard deviation.

| Analyte: | Whatman 3MM | | | Whatman 903 | | |
| --- | --- | --- | --- | --- | --- | --- |
| DBS volume: | 12.5µl | 25µl | 50µl | 12.5µl | 25µl | 50µl |
| Average Ct  (± SD) | Undetec-table | Undetec-table | 31.8  (±.14) | Undetec-table | Undetec-table | 31.6  (±.21) |

**7E. The effect of different wash conditions on the extraction efficiency of *Plasmodium falciparum* 18S rRNA from dried blood spots as assessed by a reverse-transcription PCR assay.** Each table represents an independent experiment using DBS samples with varying parasitemias (40 – 2,000 parasites/mL). A lower cycle threshold (Ct) value indicates improved efficiency. 2-Me, 2-mercaptoethanol; ETOH, ethanol; ISOH, isopropanol; SD, standard deviation.

| Volume of washes: | 750µl | 500µl | 500µl |
| --- | --- | --- | --- |
| Final 100% ETOH Wash? | YES | YES | NO |
| Average Ct  (± SD) | 27.3  (±.12) | 27.0  (±.20) | 26.8  (±.14) |

|  | Times performed | |
| --- | --- | --- |
| Wash 1 (500µl): | 2X | 1X |
| Wash 2 (500µl): | 2X | 1X |
| Average Ct  (± SD) | 34.1  (±.21) | 33.9  (±.14) |

| Wash 1: | - 2-Me | + 2-Me |
| --- | --- | --- |
| Average Ct  (± SD) | 26.0  (±.03) | 26.4  (±.30) |

| Wash 2: | 70% ETOH | 70% ETOH, 100mM NaCl, 10mM Trizma HCl pH 7.4 | 25% ETOH, 25% ISOH, 100mM NaCl, 10mM Trizma HCl pH 7.4 |
| --- | --- | --- | --- |
| Average Ct  (± SD) | 35.1  (±.14) | 34.8  (±.22) | 33.9  (±.32) |

| Wash 2: | 25% ETOH, 25% ISOH | 25% ETOH, 25% ISOH, 100mM NaCl, 10mM Trizma HCl pH 7.4 |
| --- | --- | --- |
| Average Ct  (± SD) | 27.8  (±.05) | 26.0  (±.17) |

**7F.** **The effect of different elution conditions on the elution efficiency of *Plasmodium falciparum* 18S rRNA from DNA plates as assessed with a reverse-transcription PCR assay.** Each table represents an independent experiment using DBS samples with varying parasitemias (40 – 1,000 parasites/mL). A lower cycle threshold (Ct) value improved efficiency. ISOH, isopropanol; ETOH, ethanol; TE, tris-edta solution pH 8; SD, standard deviation.

| Final spin duration (3,7000 RPM): | 5min | 10min | 20min | 2min |
| --- | --- | --- | --- | --- |
| Heat incubation: | - | - | - | 56°C 10min |
| Average Ct  (± SD) | 32.2  (±.35) | 31.8  (±.29) | 31.7  (±.24) | 30.9  (±.20) |

| Final spin duration (3,7000 RPM): | 30min | 2min |
| --- | --- | --- |
| Heat incubation: | - | 56°C 10min |
| Average Ct  (± SD) | 32.7  (±.11) | 31.9  (±.10) |

| TE Elution volume: | 50µl | 100µl | 200µl |
| --- | --- | --- | --- |
| Average Ct  (± SD) | 26.7  (±.07) | 27.6  (±.24) | 28.7  (±.08) |

| TE Elution volume: | 25µl | 50µl |
| --- | --- | --- |
| Average Ct  (± SD) | 31.1  (±.10) | 30.8  (±.20) |

| Elution buffer (50µl): | TE  (1mM EDTA) | TE-low EDTA  (0.1mM EDTA) |
| --- | --- | --- |
| Average Ct  (± SD) | 34.3  (±.41) | 34.5  (±.50) |
