# Supplementary material for: A novel method for extracting nucleic acids from dried blood spots for ultrasensitive detection of low-density Plasmodium falciparum and Plasmodium vivax infections
Source: Malar J. 2017 Sep 18;16:377. doi: 10.1186/s12936-017-2025-3 (PMC5604154; doi:10.1186/s12936-017-2025-3)
Supplement: Supplementary file 8 — Additional file 8. Testing of commercial buffers in comparison to home-made buffers on the purification efficiency of Plasmodium falciparum 18S rRNA from dried blood spots as assessed with a reverse-transcription PCR assay. Each table represents an independent experiment using DBS samples with varying parasitaemias (400 – 2,000 parasites/mL). A lower cycle threshold (Ct) value indicated improved efficiency. More information about the various buffers can be found in Additional file 4. GuSCN, guanidine thiocyanate; 2Me, 2-mercaptoethanol; ISOH, isopropanol; ETOH, ethanol; PBS, phosphate buffered saline; SD, standard deviation. [file 12936_2017_2025_MOESM8_ESM.docx]

**Additional file 8.** **Testing of commercial buffers in comparison to home-made buffers on the purification efficiency of *Plasmodium falciparum* 18S rRNA from dried blood spots as assessed with a reverse-transcription PCR assay.** Each table represents an independent experiment using DBS samples with varying parasitemias (400 – 2,000 parasites/mL). A lower cycle threshold (Ct) value indicated improved efficiency. More information about the various buffers can be found in Additional file 4. GuSCN, guanidine thiocyanate; 2Me, 2-mercaptoethanol; ISOH, isopropanol; ETOH, ethanol; PBS, phosphate buffered saline; SD, standard deviation.

| Lysis buffer: | 3M GuSCN + 16.7% ISOH | RLT-plus | RLT-plus + 16.7% ISOH | Viral | Viral + 16.7% ISOH | GenElute | GenElute + 16.7% ISOH | Purelink | Purelink + 16.7% ISOH | AL +  16.7% ISOH | ATL + 16.7% ISOH |
| --- | --- | --- | --- | --- | --- | --- | --- | --- | --- | --- | --- |
| Average Ct  (± SD) | 27.3  (± .28) | Unde-tectable | 27.9  (± 1.7) | 30.2  (± 1.1) | 33.0  (± .77) | 34.5  (± 1.1) | Unde-tectable | Unde-tectable | 29.8  (± 1.2) | Unde-tectable | Unde-tectable |

| Lysis buffer: | 3M GuSCN + 16.7% ISOH | RLT-plus + 16.7% ISOH | | | | |
| --- | --- | --- | --- | --- | --- | --- |
| Wash 1: | Lysis –  2Me | Lysis – 2Me | Sigma Wash 1 | Purelink Wash 1 | RW1 | AW1 |
| Average Ct  (± SD) | 26.0  (± .50) | 26.1  (± .38) | 27.0  (± .36) | 27.3  (± .99) | 26.0  (± .08) | 26.9  (± .47) |

| Lysis buffer: | 3M GuSCN + 16.7% ISOH | RLT-plus + 16.7% ISOH | | | | | | | | |
| --- | --- | --- | --- | --- | --- | --- | --- | --- | --- | --- |
| Wash 1: | Lysis – 2Me | Lysis – 2Me | | | | | | | | |
| Wash 2 | 25% ETOH, 25% ISOH, 100mM NaCl, 10mM Trizma HCl pH 7.4 | 25% ETOH, 25% ISOH, 100mM NaCl, 10mM Trizma HCl pH 7.4 | 25% ETOH, 25% ISOH | 70% ETOH | 70% ETOH 30% PBS | AW2 | RWT | RPE | Purelink Wash 2 | Sigma Wash 2 |
| Average Ct  (± SD) | 25.8  (± .38) | 26.1  (± .19) | 27.9  (± .65) | 26.7  (± .18) | 25.9  (± .47) | 27.2  (± .38) | Unde-tectable | 25.4  (± .26) | 26.6  (± .28) | 26.2  (± .47) |

| Lysis buffer: | 3M GuSCN + 16.7% ISOH | RLT-plus + 16.7% ISOH | | | | | |
| --- | --- | --- | --- | --- | --- | --- | --- |
| Wash 1: | Lysis – 2Me | Lysis – 2Me | | | | | |
| Wash 2 | 25% ETOH, 25% ISOH, 100mM NaCl, 10mM Trizma HCl pH 7.4 | 25% ETOH, 25% ISOH, 100mM NaCl, 10mM Trizma HCl pH 7.4 | 70% ETOH 30% PBS | 50% ETOH  50% PBS | 25% ETOH, 25% ISOH 50% PBS | RPE | Sigma Wash 2 |
| Average Ct  (± SD) | 26.2  (± .64) | 26.8  (± .30) | 26.1  (± .42) | 27.3  (± .75) | 27.0  (± .35) | 27.5  (± .47) | 28.0  (± .43) |
