# Supplementary material for: A novel method for extracting nucleic acids from dried blood spots for ultrasensitive detection of low-density Plasmodium falciparum and Plasmodium vivax infections
Source: Malar J. 2017 Sep 18;16:377. doi: 10.1186/s12936-017-2025-3 (PMC5604154; doi:10.1186/s12936-017-2025-3)
Supplement: Supplementary file 9 — Additional file 9. The growth of fungus has no effect on cycle threshold (Ct) values for detection of Plasmodium falciparum or P. vivax from dried blood spots as assessed with a reverse-transcription PCR assay for 18S rRNA. SD, standard deviation. [file 12936_2017_2025_MOESM9_ESM.docx]

**Additional file 9.** **The growth of fungus has no effect on cycle threshold (Ct) values for detection of *Plasmodium falciparum* or *P. vivax* from dried blood spots as assessed with a reverse-transcription PCR assay for 18S rRNA.** SD, standard deviation.

|  | *P. falciparum* | | *P. vivax* | |
| --- | --- | --- | --- | --- |
|  | - Fungus  (n=6) | + Fungus  (n=7) | - Fungus  (n=19) | + Fungus  (n=10) |
| Average Ct  (± SD) | 26.3  (± 1.67) | 26.8  (± 4.8) | 31.3  (± 2.4) | 28.5  (± 3.8) |
