# Supplementary material for: A novel method for extracting nucleic acids from dried blood spots for ultrasensitive detection of low-density Plasmodium falciparum and Plasmodium vivax infections
Source: Malar J. 2017 Sep 18;16:377. doi: 10.1186/s12936-017-2025-3 (PMC5604154; doi:10.1186/s12936-017-2025-3)
Supplement: Supplementary file 10 — Additional file 10. Plasmodium falciparum dried blood spot samples (400 parasites/mL) stored at room temperature in Myanmar for 3 months show similar cycle threshold (Ct) values as samples created and analysed immediately using a reverse-transcription PCR assay for P. falciparum 18S rRNA. Samples were created contemporaneously, with the only difference in storage conditions. PS, protein saver; RT, room temperature; SD, standard deviation. [file 12936_2017_2025_MOESM10_ESM.docx]

**Additional file 10.** ***Plasmodium falciparum* dried blood spot** **samples (400 parasites/mL) stored at room temperature in Myanmar for 3 months show similar cycle threshold (Ct) values as samples created and analyzed immediately using a reverse-transcription PCR assay for *P. falciparum* 18S rRNA.** Samples were created contemporaneously, with the only difference in storage conditions. PS, protein saver; RT, room temperature; SD, standard deviation.

|  | Analyzed immediately | | Stored 3 months in Myanmar | |
| --- | --- | --- | --- | --- |
|  | Whatman 3MM | Whatman 903 PS | Whatman 3MM | Whatman 903 PS |
| Average Ct  (± SD) | 30.0  (± .85) | 29.8  (± .21) | 29.7  (± .30) | 29.4  (± .34) |
