# Supplementary material for: A novel method for extracting nucleic acids from dried blood spots for ultrasensitive detection of low-density Plasmodium falciparum and Plasmodium vivax infections
Source: Malar J. 2017 Sep 18;16:377. doi: 10.1186/s12936-017-2025-3 (PMC5604154; doi:10.1186/s12936-017-2025-3)
Supplement: Supplementary file 11 — Additional file 11. Representative cycle threshold (Ct) values for Plasmodium falciparum 18S rRNA and human actin RNA from dried blood spot standards (n=4) as assessed with a reverse-transcription PCR assay. SD, standard deviation. [file 12936_2017_2025_MOESM11_ESM.docx]

**Additional file 11.** **Representative cycle threshold (Ct) values for *Plasmodium falciparum* 18S rRNA and human *actin* RNA from dried blood spot standards (n=4) as assessed with a reverse-transcription PCR assay.** SD, standard deviation.

| Parasites/mL | 250,000 | 50,000 | 10,000 | 2,000 | 400 | 80 | 16 | 0 |
| --- | --- | --- | --- | --- | --- | --- | --- | --- |
| Average Ct  18S rRNA (± SD) | 18.5  (± .21) | 20.9  (± .34) | 23.0  (± .32) | 25.5  (± .42) | 27.3  (± .62) | 31.7  (± .87) | 31.3*  (± .60) | Unde-tectable |
| Average Ct  human *actin* (± SD) | 23.5  (± .04) | 23.4  (± .28) | 23.3  (± .33) | 23.4  (± .17) | 23.1  (± .27) | 23.0  (± .21) | 22.9  (± .20) | 22.3  (± .39) |

* Only 3/4 DBS showed positivity at this concentration
