# Supplementary material for: A novel method for extracting nucleic acids from dried blood spots for ultrasensitive detection of low-density Plasmodium falciparum and Plasmodium vivax infections
Source: Malar J. 2017 Sep 18;16:377. doi: 10.1186/s12936-017-2025-3 (PMC5604154; doi:10.1186/s12936-017-2025-3)
Supplement: Supplementary file 12 — Additional file 12. The range, median, mean and standard deviation for human actin cycle threshold (Ct) values from a field survey in Myanmar (n=165) as assessed with a reverse-transcription PCR assay. SD, standard deviation. [file 12936_2017_2025_MOESM12_ESM.docx]

**Additional file 12.** **The range, median, mean and standard deviation for human *actin* cycle threshold (Ct) values from a field survey in Myanmar (n=165) as assessed with a reverse-transcription PCR assay.** SD, standard deviation.

|  | Range | Median | Mean | Standard deviation |
| --- | --- | --- | --- | --- |
| Actin Ct value | 18.9 – 24.5 | 22.0 | 21.9 | 1.2 |
